# Early recovery after fast-track Oxford unicompartmental knee arthroplasty: 35 patients with minimal invasive surgery

**DOI:** 10.3109/17453674.2012.657578

**Published:** 2012-02-08

**Authors:** Stig Munk, Jesper Dalsgaard, Karin Bjerggaard, Ina Andersen, Torben Bæk Hansen, Henrik Kehlet

**Affiliations:** ^1^Department of Orthopaedics, Holstebro Regional Hospital; ^2^The Lundbeck Center for Fast-Track Hip and Knee Arthroplasty, Rigshospitalet, Copenhagen University; ^3^Department of Physiotherapy, Holstebro Regional Hospital; ^4^Department of Mathematics, Aarhus University, Aarhus; ^5^Orthopaedic Research Unit, Hospital Unit West; ^6^Section of Surgical Pathophysiology, Rigshospitalet, Copenhagen University Hospital, Denmark

## Abstract

**Background and purpose:**

After total knee arthroplasty with conventional surgical approach, more than half of the quadriceps extension strength is lost in the first postoperative month. Unicompartmental knee arthroplasty (UKA) operated with minimally invasive surgery (MIS) results in less operative trauma. We investigated changes in leg-extension power (LEP) in the first month after MIS Oxford UKA and its relation to pain, knee motion, functional performance, and knee function.

**Patients and methods:**

In 35 consecutive Oxford UKA patients, LEP was measured 1 week before and 1 month after surgery together with knee motion, knee swelling, the 30-second chair-stand test, and Oxford knee score. Assessment of knee pain at rest and walking was done using a visual analog scale.

**Results:**

30 patients were discharged on the day after surgery, and 5 on the second day after surgery. LEP and functional performance reached the preoperative level after 1 month. Only slight postoperative knee swelling was observed with rapid restoration of knee flexion and function. A high level of pain during the first postoperative night and day fell considerably thereafter. None of the patients needed physiotherapy supervision in the first month after discharge.

**Interpretation:**

Fast-track MIS Oxford UKA with discharge on the day after surgery is safe and leads to early recovery of knee motion and strength even when no physiotherapy is used.

After total knee arthroplasty (TKA), more than half of the preoperative quadriceps strength is lost in the first month after surgery (Mizner et al. 2005). TKA surgery with minimally invasive surgery (MIS) may lead to less postoperative pain, shorter length of stay in hospital, and greater knee flexion (Chen et al. 2006), and the MIS approach may result in better outcome with regard to maintaining extensor strength than the conventional surgical approach (Kim et al. 2010). In unicompartment knee arthrosis, the Oxford Group recommend unicompartmental knee arthroplasty (UKA) operated with MIS, since recovery is twice as fast as after the conventional approach and damage to the soft tissue of the joint is greatly diminished (Price et al. 2001). Althrough fast-track UKA with length of stay of about 1.5 days has been described (Reilly et al. 2005), there have been few reports on quadriceps strength after UKA (Mancher et al. 2002) and to our knowledge there have been no reports on early leg-extension power (LEP) after UKA.

We studied changes in LEP within the first month after fast-track MIS Oxford Phase III medial UKA in relation to pain, knee motion, functional performance, knee function, and the need for physiotherapy after discharge.

## Patients and methods

### Subjects

This prospective study included 39 consecutive patients who were scheduled for primary unilateral Oxford medial UKA (Oxford Knee Phase III; Biomet Ltd., Bridgend, UK) at the Department of Orthopedics, Holstebro Regional Hospital from May 2010 through January 2011. The patients were included according to the criteria described by the Oxford Group (Price et al. 2001).

All patients were operated under spinal or general anesthesia by one of two surgeons with good experience of MIS and Oxford UKA. Cefuroxime (1.5 g) was administered intravenously before surgery and tranexamic acid (1 g) was administered intravenously at the end of surgery. Local infiltration analgesia (LIA) was used intraoperatively (Kerr and Kohan 2008). No drains were used. At the end of surgery, an elastic compression bandage was used from the toes to the mid-thigh (Andersen et al. 2008) and a cooling device was also used (Cryo/cuff; Aircast Europe GmbH, Hässelby, Sweden).

All patients followed the same fast-track program for elective UKA that had been used at the hospital since 2009 (Husted et al. 2008). It included preoperative multidisciplinary education and well-defined optimized multimodal pain treatment for 1 week including 1 g paracetamol 6-hourly, 200 mg celecoxib 12-hourly, 300 mg gabapentin in the morning and 600 mg in the evening, and oxycodone (5 mg) for pain rescue.

The patients were ambulatory 2–4 h after surgey. Low molecular heparin (5,000 U) was given 6–8 hours after surgery and was continued daily until discharge. The compression bandage was removed in the morning on the day after surgery, and free active knee movement was allowed. The patients were discharged according to well-defined discharge criteria and were given written advice on potential problems with a 24-h emergency contact telephone number. They were also contacted by telephone on the day after discharge. All patients were seen at the outpatient clinic after 1 and 2 weeks, and at the outpatient physiotherapy unit after 4 weeks to re-assess and adjust home exercises.

### Leg-extension power

The maximal leg-extension power (LEP) was measured 1 week before and 1 month after surgery using a leg extension power rig (Bio-Med International, Nottingham, UK) as described by Barker et al. (2011); this measures the power in a single leg extension. The patient is seated in the rig with the most comfortable knee and hip flexion, with a seat belt attached across the hips and with the foot of the operated leg on the dynamometer pedal. The patient is then instructed to extend the leg as forcefully and quickly as possible, and strong verbal encouragement is provided during the contractions. 5 contractions are performed, and the highest value of the 5 is used. Leg-extension power is expressed as power (in Watts) per kg of body mass. LEP measurements were obtained for both legs.

### Knee motion

Active range of knee motion (ROM) was measured before surgery, on days 1, 7, and 14, and 1 month after surgery using a standardized manual (Rothstein and Miller 1983). The patients were supine during the measurements. A 360-degree plastic goniometer with 30-cm movable arms, scaled in 1 degree, was used. 2 measurements were done and the one with the highest value was used.

### Knee swelling

Knee swelling was measured before surgery, on days 1, 7, and 14, and 1 month after surgery. With the patient relaxed in supine position, the circumference of the extended knee was measured 1 cm proximal to the base of the patella (Ross and Worrell 1998). 2 measurements were done and the mean value was used. Changes were quantified as relative changes from baseline values.

### Functional performance

Functional performance was evaluated 1 week before and 1 month after surgery by the 30-second chair-stand test. The test measures how many times the patient is able to rise from a chair and sit down again in 30 seconds. The test has been shown to be a reliable and valid indicator of lower body strength (Jones et al. 1999). Changes were quantified as number of repetitions in 30 seconds.

### Knee function

Knee status was assessed by using the summed score from the Oxford knee score (OKS) questionnaire (Dawson et al. 1998). The OKS can vary from 0 to 48, where 48 is the best possible outcome.

### Pain

Assessment of pain was done using a 100-mm visual analog scale (VAS). A pain diary was given to the patients 1 week before surgery, and they were asked to note pain at rest and during walking 3 times a day as well as pain at night preoperatively, from day 0 to day 14 and on day 30. Use of strong and weak opioids was recorded for the same period.

### Statistics

Box plots were prepared to illustrate the results. The box plots show the median, interquartile range (box), range (whiskers), and outliers (data more than 1.5 times the interquartile range from the box). Correlations were calculated as Spearman's rho. The program R, version 2.10.0, was used for all statistical calculations.

## Results

39 patients were scheduled for surgery. 1 patient was excluded because of preoperative cognitive dysfunction and 2 refused to participate. 1 patient with heart disease and anticoagulation therapy developed tense hemarthrosis and subcutaneous wound hematoma postoperatively and was excluded because of deep infection diagnosed 2 weeks after surgery. No other complications were registered. Thus, 35 patients (18 men) with a mean age of 66 (51–81) years completed the study. 13 patients had bilateral knee osteoarthritis and 5 had a prosthesis in the other knee.

30 patients were discharged on the day after surgery—after training and after measurement of knee motion and swelling. 5 patients were discharged on the second postoperative day because of postoperative pain or side effects of pain medication. None of the patients had physiotherapy consultations before the 1-month visit and none were referred for physiotherapy.

**Figure 1.**
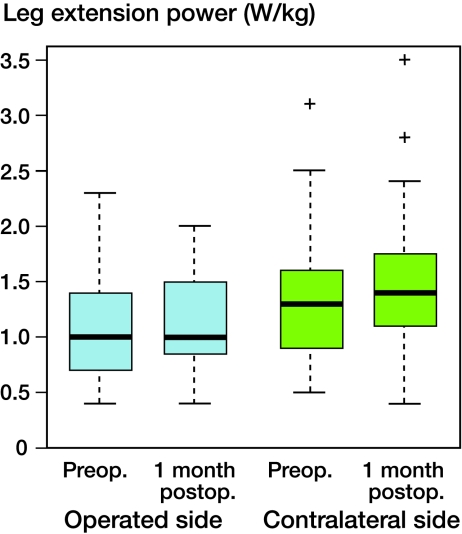
Leg-extension power preoperatively and after 1 month, in operated knees (light blue) and contralateral knees (green). Boxes represent the interquartile range (IQR), whiskers the range, and + are outliers (> 1.5 x IQR).

**Figure 2.**
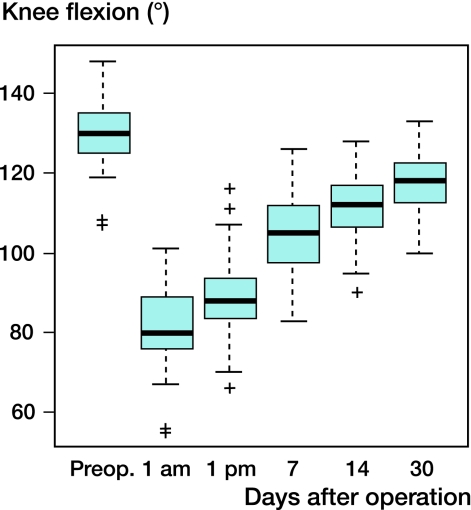
Knee flexion during the first postoperative month.

**Figure 3.**
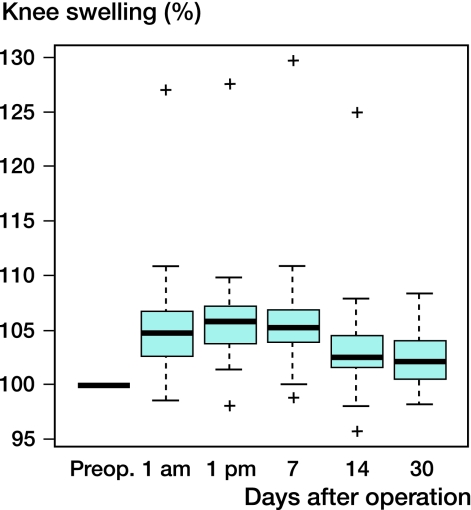
Postoperative change in knee swelling.

**Figure 4.**
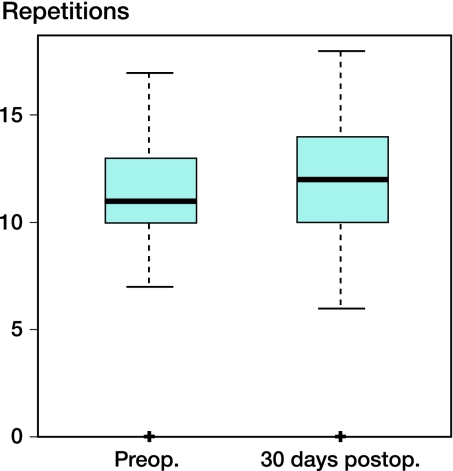
30-second sit-to-stand test before and after UKA.

**Figure 5.**
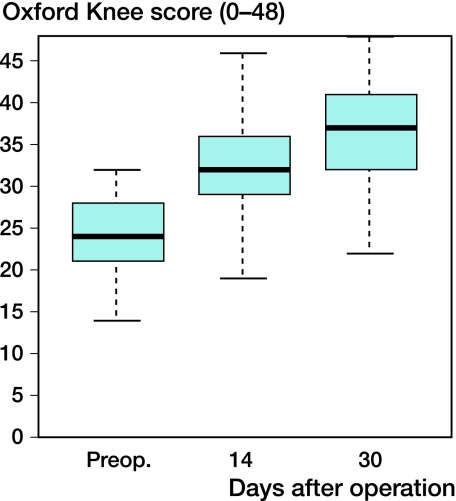
Oxford knee score before and after UKA.

Before surgery, LEP in the operated knees was 1 (0.4–2.3) W/kg, and after 1 month it was 1 (0.4–2.0) W/kg. The corresponding values in the contralateral knees were 1.3 (0.5–3.1) W/kg and 1.4 (0.4–3.5) W/kg (Figure 1). Before surgery, knee flexion was 130° (107–148) and after 1 month it was 118° (100–133) (Figure 2). Before surgery, knee extension was 3° (-8 to 7) and after 1 month it was 7° (4–17). Knee circumference increased to 6% at most but fell to 2% after 1 month (Figure 3). Sit-to-stand performance was 11 (0–17) times in 30 seconds before surgery and 12 (0–18) times 1 month after surgery (Figure 4). There was a statistically significant correlation between LEP and the 30-second stand-to-sit test preoperatively (r = 0.32, p = 0.05) and 1 month after surgery (r = 0.45, p = 0.007) but not between LEP and pain or knee flexion. There was a positive correlation before and after surgery between LEP and OKS, but it was not statistically significant (r = 0.32, p = 0.3; and r = 0.21, p = 0.2). OKS increased from 24 (14–32) preoperatively to 37 (22–48) 1 month after surgery (Figure 5).

**Figure 6.**
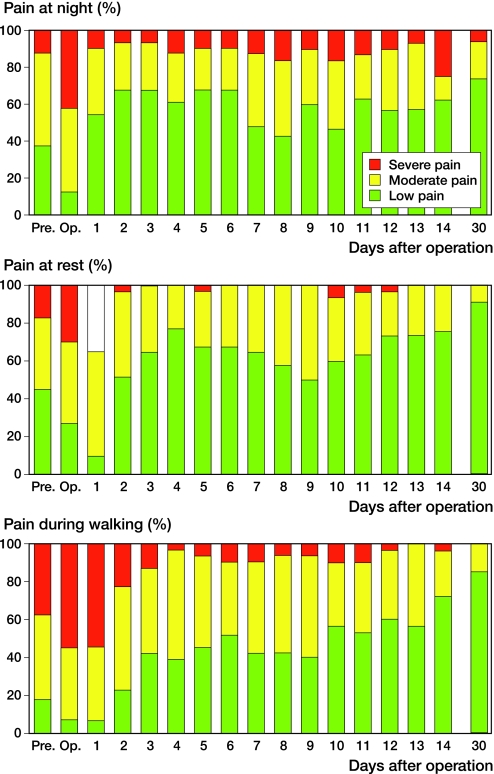
Proportions of patients who reported low (0–29 mm on a 100-mm VAS), moderate (30–59 mm), or severe (60–100 mm) pain at night, at rest, and during walking.

**Figure 7.**
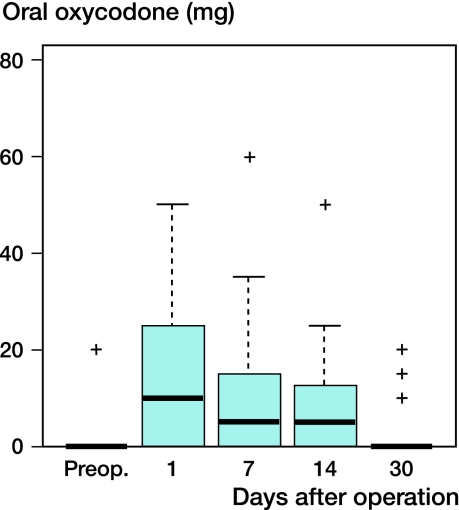
Use of oral oxycodone during the first postoperative month after UKA.

Patient-reported pain (with correction for missing data) was as follows. Preoperatively, 15 of 24 patients reported moderate or severe pain at night, 14 of 27 reported moderate or severe pain at rest, and 24 of 29 reported moderate or severe pain during walking. The night after surgery, 27 of 31 patients experienced moderate or severe pain. 28 of 31 patients reported moderate or severe pain at rest on the day after surgery whereas 29 of 31 reported moderate or severe pain during walking. 1 month after surgery, 9 of 35 patients experienced moderate or severe pain at night, 3 of 35 experienced moderate pain at rest, 5 of 35 experienced moderate pain during walking, and no patients had severe pain at rest or during walking (Figure 6). Use of oxycodone was mean 10 (10–50) mg on the day after surgery and 0 (0–20) mg after 1 month (Figure 7).

## Discussion

Minzer et al. (2005) found that 1 month after TKA, quadriceps muscle strength decreased by 62% relative to the preoperative level. Mancher et al. (2002) found an increase in strength of the quadriceps muscle 18 months after UKA in both operated and non-operated legs, but they did not focus on early recovery. We found that 1 month after Oxford medial UKA with minimally invasive surgery, the average LEP in both legs and the functional performance were comparable to those at the preoperative level. This is different from TKA patients at our own institution, where we have found that LEP after 1 month is 30% lower than in the present study (Aalund P K, personal communcation).

Holm et al. (2010) found that there was a correlation between knee swelling and loss of knee-extension strength early after TKA with conventional approach, with a 12% increase in the average circumference of the knee joint at discharge (2.4 days after surgery). We found maximum knee swelling to be 6% in the first week after UKA. The Oxford Group (Reilly et al. 2005) used a pressure bandage for 5 days to limit the degree of flexion because of a suspected increased risk of hemarthrosis. We removed the bandage before 24 hours and allowed free active knee flexion, which led to rapid restoration of range of motion with only slight knee swelling, and none had less than 100° of flexion at 4 weeks.

In the accelerated recovery protocol of Reilly et al. (2005), the 41 patiens were given an extension splint to help mobilization of the knee 2–4 hours after surgery. The average discharge time was 1.5 days. In the present study, we did not use an extension splint. It does not seem to have delayed mobilization, and the average discharge time was 1.1 days.

Local infiltration analgesia (LIA) gives effective pain relief with the possibility of early mobilization. In a double-blind study, Essving et al. (2009) found lower morphine consumption and pain relief postoperatively with LIA. They used an intraarticular LIA catheter, which was removed after 24 h, and the median length of stay was 1 (1–6) days.

In the present study, the patients reported a high level of pain during the first postoperative night and the next day, but the level of pain fell sharply thereafter. Andersen et al. (2009) reported that 40% of patients had moderate or severe pain when walking, 1 month after TKA, while none of the patients in the present study reported severe pain and only 14% reported moderate pain during walking 1 month after UKA.

Our results confirm that fast-track Oxford UKA with discharge on the day after surgery is a safe procedure (Reilly et al. 2005, Essving et al. 2009). A pressure bandage need not be used for more than 24 h unless in patients with increased risk of intra-articular bleeding. Due to the high level of pain and use of strong opioids in the initial period after surgery, we would not recommend Oxford UKA as an outpatient procedure unless analgesia is improved. In this context, a preoperative dose of methylprednisolone may be an option, as shown in TKA (Lunn et al. 2011). We found rapid recovery as assessed by the Oxford knee score, and the lack of supervised physiotherapy does not appear to have been a disadvantage to the patients regarding early knee flexion and knee function.
